# Efficacy and Safety of Microsurgery in Interdisciplinary Treatment of Sarcoma Affecting the Bone

**DOI:** 10.3389/fonc.2019.01300

**Published:** 2019-11-26

**Authors:** Johannes Zeller, Jurij Kiefer, David Braig, Oscar Winninger, David Dovi-Akue, Georg W. Herget, G. B. Stark, Steffen U. Eisenhardt

**Affiliations:** ^1^Department of Plastic and Hand Surgery, Faculty of Medicine, Medical Center—University of Freiburg, University of Freiburg, Freiburg, Germany; ^2^Plastic and Reconstructive Surgery, Sir Charles Gairdner Hospital, Perth, WA, Australia; ^3^Division of Hand, Plastic and Aesthetic Surgery, University Hospital, LMU Munich, Munich, Germany; ^4^Department of Orthopedics and Trauma Surgery, Faculty of Medicine, Medical Center—University of Freiburg, University of Freiburg, Freiburg, Germany

**Keywords:** soft tissue sarcoma (STS), microsurgery, interdisciplinary/multidisciplinary, bone sarcoma, free tissue transfer

## Abstract

**Background:** Sarcomas are tumors of mesenchymal origin with high variation in anatomical localization. Sarcomas affecting the bone often require an interdisciplinary resection and reconstruction approach. However, it is critical that microsurgical reconstruction strategies do not negatively impact tumor safety and overall survival, as limb salvage is only the secondary goal of tumor surgery. Here, we analyzed the efficacy and safety of microsurgery in interdisciplinary treatment of sarcoma affecting the bone.

**Patients and Methods:** We performed a retrospective chart review of all patients treated for soft-tissue and bone sarcoma at the senior author's institution with a focus on bone affection and microsurgical reconstruction between 2000 and 2019. This particular subgroup was further investigated for tumor resection status, 5-year survival rate, length of hospital stay, as well as overall complication and amputation rates.

**Results:** Between 2000 and 2019, 803 patients were operated for sarcoma resection and reconstruction by the Department of Plastic and Hand Surgery. Of these, 212 patients presented with sarcoma of the extremity affecting the bone. Within this subgroup, 40 patients required microsurgical reconstruction for limb salvage, which was possible in 38 cases. R0 resection was achieved in 93.8%. The 5-year survival was 96.7%, and the overall complication rate was 25%, of which 40% were microsurgery associated complications.

**Conclusion:** Safe and function-preserving treatment of soft-tissue and bone sarcoma is challenging. Primary reconstruction with microsurgical techniques of sarcoma-related defects enables limb-sparing and adequate oncosurgical cancer treatment without increasing the risk for local recurrence or prolonged hospital stay. The treatment of sarcoma patients should be reserved to high-volume centers with experienced plastic surgeon embedded in a comprehensive treatment concept.

## Introduction

Sarcomas are a rare and complex entity of tumors arising from tissues of mesodermal origin. With the mesoderm forming both smooth and skeletal muscle, connective tissue, fat, and synovial tissue, sarcomas are not restricted to a specific anatomical location. This highly diverse group of malignancies accounts for <1% of all malignant disorders in adults, yet the current WHO Classification of Diseases and Oncology subdivides sarcomas into more than 100 histologic subtypes (1, 2). Therefore, the variety in localization and histological findings presents a significant challenge for the attending surgeon. For most subtypes, the mainstay of treatment is the surgical excision of the sarcoma. Innovations in reconstructive surgery and interdisciplinary treatment led to safe limb-sparing cancer treatment with amputation rates under ten percent over the last years (3, 4). Improved reconstructive options embedded in a multimodal treatment expanded limb-salvage rates to over 95% of cases (5). Furthermore, recent studies evoke a shift in the paradigm on resection margins in soft tissue sarcoma, with long term safe results in limited sarcoma resection (6). In cases where primary closure after oncological resection is not achievable, microsurgical reconstruction with free tissue transfers allows for sufficient soft tissue coverage and preservation of limb function.

At the Medical Center—University of Freiburg, Germany, patients with localized soft tissue sarcoma are primarily treated by the Department of Plastic and Hand Surgery. Most patients with bone sarcoma or soft tissue sarcoma affecting the bone require an interdisciplinary surgical approach and are, therefore, treated together with the Department of Orthopedics and Trauma Surgery. To optimize all aspects of the cancer treatment, e.g., (neo-)adjuvant and intraoperative radiation therapy, or chemotherapy, every case is discussed in an interdisciplinary tumor board.

Here, we reviewed data from nearly 20 years of multidisciplinary and single-center management of patients with sarcomas regarding surgical treatment modalities and outcome. We further analyzed our results for a subgroup of patients treated in curative intent with bone sarcoma or soft tissue sarcoma affecting the bone, who received microsurgical reconstruction with free tissue transfer.

## Materials and Methods

We performed a retrospective chart review to analyze all patients treated for sarcoma between January 2000 and July 2019. Within this cohort, we further investigated patients who required complex microsurgical reconstruction for bone sarcoma and soft-tissue sarcoma infiltrating the bone or affecting bone stability after resection for bone sarcoma and soft-tissue sarcoma infiltrating the bone or affecting bone stability after resection. These patients were treated in an interdisciplinary approach by the Department of Plastic and Hand Surgery and the Department of Orthopedics and Trauma Surgery, Medical Center—University of Freiburg. Patients with dermatofibrosarcoma protuberans, pleomorphic dermal sarcoma, and sarcomas of the retroperitoneum were excluded from this study. To reduce heterogeneity, we also excluded sarcoma cases treated with other surgical disciplines, i.e., thoracic or vascular surgery. Clinical notes and pathology reports were reviewed for patient-related data regarding the operative procedure, demographic information, localization of the tumor, histopathological diagnosis, and resection status. Surgical details on flap selection, complications, and further microsurgical information were analyzed from the operative reports.

Statistical analysis was performed using GraphPad Prism v9 for Mac, GraphPad Software, La Jolla California USA (www.graphpad.com). *Fisher's* exact test, *Mann-Whitney* rank-sum, and unpaired *t*-test (*Student's* test), respectively, were used for statistical analysis. A *p*-value < 0.05 was regarded as statistically significant.

## Results

### Patient Characteristics

A total of 803 patients with either soft tissue sarcoma, bone sarcoma, or soft tissue sarcoma infiltrating bones were treated with curative intent between January 2000 and July 2019 by the Department of Plastic and Hand Surgery. Overall, the distribution of sex for our patient population was 46% female and 54% male with a mean age of 58.6 ± 18 years. Seven cases were excluded due to incomplete or missing data. The further analyzed subgroup of patients with bone-associated sarcoma who received microsurgical reconstruction accounted for 48 patients. The mean age of this cohort was 54.2 ± 21.7 years (mean ± SD) with 21 female (43.8%) and 27 male patients (56.2%). Patients of this subgroup underwent tumor surgery, including resection of bone and received microsurgical reconstruction of bone and soft tissue to preserve limb functionality.

### Histopathological Characteristics

The anatomical distribution of sarcomas was analyzed based on operative and pathological reports and pre-operative imaging. Overall, sarcomas most frequently affected the lower extremities (51.8% of all patients). Upper extremities, trunk, and head and neck accounted for 19.8, 17.2, and 11.2%, respectively. The most common anatomic location within patients receiving microsurgical coverage was the lower extremities in 32 cases (66.7%). For sarcoma infiltrating bones of the upper extremity, eight patients (16.7%) required microsurgical reconstruction. Sarcoma with bone affection and microsurgical defect coverage located in the trunk comprised of four patients (8.3%), and head and neck sarcoma accounted for four patients (8.3%). In all cases, sarcoma either infiltrated the bone or associated bone tissue had to be resected to ensure cancer-free margins. In nine patients, replacement of the joint with tumor prosthesis had to be performed to achieve limb-salvage, and in 28 patients, extensive bone resection (>3 cm) was performed to ensure tumor-free margins. In 11 cases, limited bone resection was considered sufficient. In patients with oncosurgical bone resections following free tissue transfer, microscopically free margins were achieved in 45 cases (93.8%). Three patients were identified as R1 with microscopically residual tumor cells. In two cases, revision surgery had to be performed to achieve tumor-free margins. In one case, the affected limb was amputated to achieve tumor clearance. Overall, sarcomas were located in the limbs in 40 cases. For these patients, limb salvage was achieved in 90%.

The histopathological diagnosis was based on the WHO classification. Osteosarcoma (nine patients), fibrosarcoma (eight patients), and undifferentiated pleomorphic sarcoma (nine patients) were the most common histologic categories. These entities accounted for over 50% of the cases (Table 1).

**Table 1:** Demographic characteristics of sarcoma patients and outcome information.

**Age in years**	**Sex**	**Entity**	**Grading**	**Microsurgical procedure**	**Resection status**	**Local recurrence**	**Radiotherapy**	**Flap revisions**	**Flap loss**
42	w	Leiomyosarcoma	G2/3	Fibula	R0	No	Neo-adjuvant	None	–
65	m	Undifferentiated liposarcoma	G3	ALT	R1	No	Neo-adjuvant	None	–
83	m	Undifferentiated pleomorphic sarcoma	G3	ALT	R0	No	Adjuvant	None	–
52	w	Fibrobrous synovial sarcoma	G2	ALT	R0	No	Neo-adjuvant	Venous	–
60	m	Undifferentiated pleomorphic sarcoma	G3	ALT	R1	No	Neo-adjuvant	None	–
56	m	myxofibrosarcoma	G2	ALT	R0	No	No	None	–
72	w	Osteosarcoma	G3	ALT	R0	No	Neo-adjuvant	None	–
29	m	Osteosarcoma	G3	Gracilis	R0	No	No	None	–
17	w	Osteosarcoma	G3	Fibula	R0	No	Neo-adjuvant	Arterial	–
59	w	Sarcoma NOS	G3	Latissimus dorsi	R0	No	Adjuvant	None	–
83	m	Undifferentiated pleomorphic sarcoma	G3	ALT	R0	No	Adjuvant	None	–
52	m	Leiomyosarcoma	G2/3	ALT	R0	No	Adjuvant	None	–
17	w	Osteosarcoma	G3	Gracilis	R0	No	No	None	–
29	m	Osteosarcoma	G3	Gracilis	R0	No	No	None	–
37	m	Undifferenti ated pleomorphic sarcoma	G3	Rectus abdominis	R0	Yes	No	None	–
76	m	Undifferentiated pleomorphic sarcoma	G3	Gracilis	R0	No	Neo-adjuvant	None	–
50	m	Synovial sarcoma	G2	Latissimus dorsi	R0	No	Neo-adjuvant	None	–
59	w	Sarcoma NOS	G3	ALT	R0	No	No	None	–
36	m	Undifferentiated pleomorphic sarcoma	G3	Rectus abdominis	R0	Yes	No	None	–
63	w	Undifferentiated pleomorphi c sarcoma	G3	Latissimus dorsi	R0	No	Adjuvant	None	–
74	w	Sarcoma NOS	G3	Rectus abdominis	R0	No	Adjuvant	None	–
81	m	myxofibrosarcoma	G3	Latissimus dorsi	R0	No	Adjuvant	None	–
39	m	myxoid liposarcoma	G1	Parascapular	R0	No	Neo-adjuvant	None	–
88	m	Undifferentiated pleomorphic sarcoma	G3	Radialis	R0	No	No	None	–
77	m	Undifferentiated pleomorphic sarcoma	G3	ALT	R0	Yes	Adjuvant	None	–
68	m	Undifferentiated spindl e cell sarcoma	G3	Latissimus dorsi	R0	No	Adjuvant	None	–
10	m	Alveolar rhabdomyosarcoma	G3	Parascapular	R0	No	No	None	–
71	w	Liposarcoma	G2	Parascapular	R0	No	No	None	–
71	w	Fibrobrous synovial sarcoma	G2	ALT	R1	No	Adjuvant	None	–
52	m	Leiomyosarcoma	G2/3	ALT	R0	No	Adjuvant	None	–
90	m	Sarcoma NOS	G3	ALT	R0	No	No	None	–
74	w	myxofibrosarcoma	G1	ALT	R0	Yes	No	None	–
46	w	Sarcoma NOS	G3	Rectus abdominis	R0	Yes	Adjuvant	None	–
48	m	Angi osarcoma	G2	Latissimus dorsi	R0	No	Adjuvant	None	–
35	m	Alveolar rhabdomyosarcoma	G3	ALT	R0	No	Adjuvant	None	–
85	w	Sarcoma NOS	G3	Latissimus dorsi	R0	No	No	None	–
53	w	Fibrosarcoma	G3	Rectus	R0	No	No	None	–
56	m	Fibrosarcoma	G3	Lat issimus dorsi	R0	Yes	No	Arterial	–
21	m	Osteosarcoma	G3	ALT	R0	No	No	None	–
21	m	Osteosarcoma	G3	ALT	R0	No	No	None	–
72	w	Dedifferentiated chondrosarcoma	G3	ALT	R0	No	Adjuvant	None	–
77	m	Dedifferentiated chondrosarcoma	G3	Fibula	R0	No	Adjuvant	None	–
65	w	Osteosarcoma	G3	ALT	R0	No	Neo-adjuvant	None	–
44	w	Synovial sarcoma	G2	Rectus abdominis	R0	No	Adjuvant	Arterial	Yes
23	w	Rhabdomyosarcoma	G3	Latissimus dorsi	R0	No	No	Venous	–
15	m	Osteosarcoma	G3	Fibula	R0	No	Neo-adjuvant	None	–
59	w	myofibroblastic sarcoma	G3	ALT	R0	No	Adjuvant	None	–
49	w	Fibrosarcoma	G3	Fibula	R0	No	No	None	Partial

*Grading: G1 Well differentiated (Low grade), G2 Moderately differentiated (Intermediate grade), G3 Poorly differentiated (High grade). ALT, Anterolateral thigh flap*.

### 5-Year Survival and Remission Status

Within the observed period, 30 out of 48 patients revealed to be free of sarcoma. Six patients developed local recurrence, and 12 patients presented with distant metastasis. Five years after tumor resection and microsurgical coverage, 30 patients were alive. In 18 cases, tumor surgery was performed within the last 5 years of the study period. Of these patients, 17 were alive. One patient died due to the progression of the disease.

### Microsurgical Procedures

The microsurgical procedures performed on sarcoma patients are described hereafter. The most frequently utilized flap was the fasciocutaneos anterolateral thigh flap (ALT), which was utilized in 20 cases (41.7%). The latissimus dorsi flap was the most common muscle flap used for microsurgical reconstruction (nine patients, 18.8%). Minor complications occurred in seven cases (twice seroma and five times wound dehiscence). Five patients had to undergo revision surgery due to insufficient blood flow (three times arterial and twice venous congestion). In two case, total flap loss occurred (Table 1).

The following four cases demonstrate study patients treated in an interdisciplinary approach. Each case represents one of the main challenges encountered with bone involvement: prophylactical osteosynthesis in patients with a high risk for secondary fractures, stabilization, and bone bridging with free autografts in primary bone instability, and coverage of tumor endoprosthesis.

**Figure 1:**
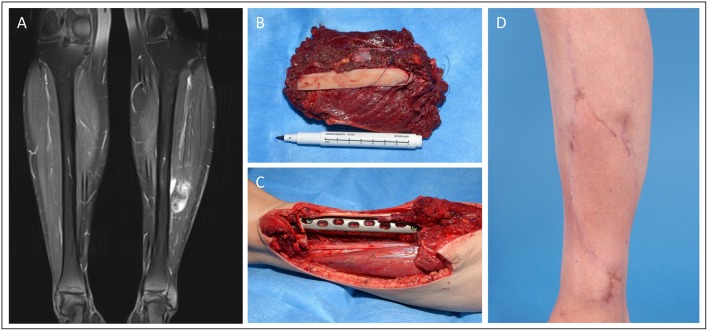
Patient operated in an interdisciplinary approach for synovial sarcoma of the lower extremity. **(A)** Pre-OP MRI of the lower extremity with visible mass in the left lower leg. **(B)** Excised tumor tissue with resected fibula segment. **(C)** Tumor bed with prophylactic plate osteosynthesis on tibia. **(D)** Clinical presentation in the 6 months follow-up.

Case 1 (Figure 1) shows the case of a 52-year old female patient presenting with a G2 synovial sarcoma in the left lower leg (ICD-O M-9040/3., UICC stage IIIA). Sarcoma was infiltrating the posterior compartment of the lower leg and showed contact to both fibula and tibia in the pre-operative imaging. To ensure tumor-free resection margins, the surgical approach included segmental resection of the fibula and wide osteotomy of the dorsolateral cortex of the tibia. Then, a locking compression plate was used to prevent secondary fracture. The transfer of a free ALT flap was performed to cover the resulting defect (8 × 16 cm).

**Figure 2:**
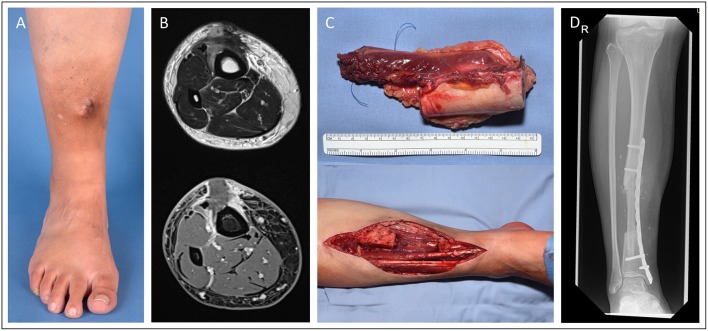
Patient presenting with a tibia-infiltrating leiomyosarcoma. Resulting primary bone defect was bridged by fibula-pro-tibia operation in an interdisciplinary approach. Pre-OP clinical **(A)** and MRI **(B)** presentation of the tumor. **(C)** Excised tumor tissue with affected tibia segment and lower leg during the resection. **(D)** Beginning tibialization of the fibula graft in the 8 months follow-up x-ray.

In Figure 2, we demonstrate a 42-year-old female patient suffering from a leiomyosarcoma of her lower leg (ICD-O M-8890/3. ypT1, L0, V0, PN0. G2-3, UICC stage II). First, the affected lower leg received neoadjuvant radiation therapy with a total dose of 50 Gy applied. Then, we performed a limb-sparing, wide resection to ensure tumor-free margins. The osteotomy and osteosynthesis of the tibia were performed by the Department for Orthopedics and Trauma Surgery. The resulting bone defect of 8 cm length and soft-tissue defect (7 × 14 cm) was reconstructed with a free osteocutaneous fibula flap.

**Figure 3:**
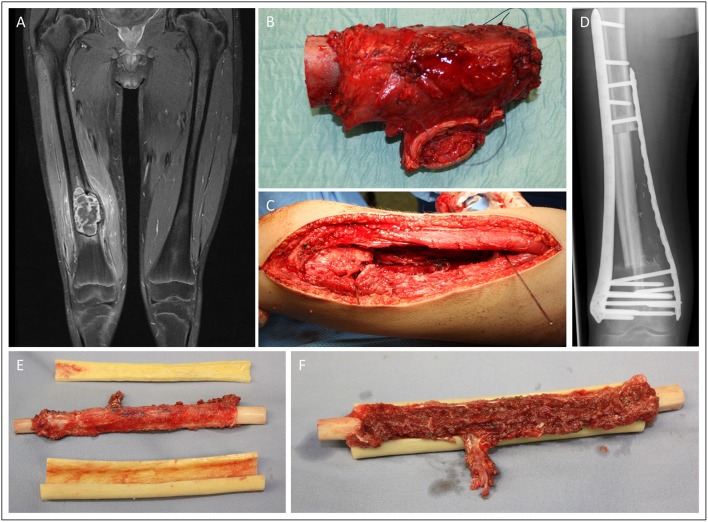
Fifteen-year old male patient presenting with osteosarcoma in the distal femur. Femoral reconstruction was performed with a free fibula graft combined in an allograft as described by Capanna. Pre-OP MRI presentation of the sacroma mass in the distal femur **(A)**. Intraoperative images of the resected tumor (13 cm length) **(B)** and the resulting femoral defect **(C)**. X-ray of the result in the 2 month follow-up **(D)**. **(E)** and **(F)** demonstrate the intraoperative preparation of the microvascular free fibular autograft supported by a peripheral massive allograft shell.

In case 3 (Figure 3), we demonstrate a 15-year old male patient suffering from extensive osteosarcoma in the distal femoral bone (9.1 cm in longitudinal length; ypT2, L0. V0. Pn0). Neoadjuvant chemotherapy was effective and reduced vital tumor cells by 90% while tumor size did not differ to pre-chemotherapy imaging. Consecutively, a 13-cm long segment of the distal femur was resected. For femoral reconstruction, free a fibula graft was harvested from the left limb and used as an intramedullary vascularized graft combined with an allograft as described by Capanna et al. (7) and Ceruso et al. (8). The osteosynthesis was performed using a locking compression plate and a less invasive stabilization system (LISS) plate. Resection margins were microscopically free of tumor cells. The patient presented with normal gait and function and without any difference in leg length 2 months post-operatively.

**Figure 4:**
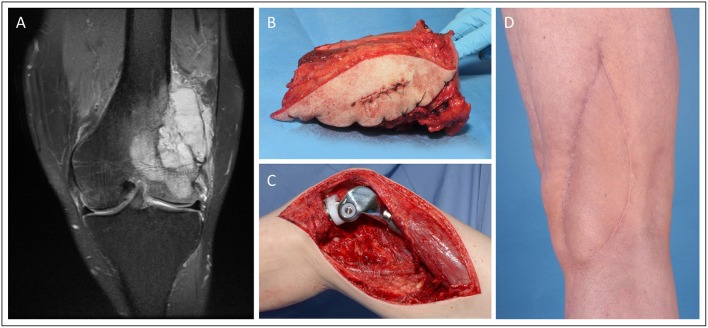
Knee reconstruction with tumor prosthesis and microsurgical soft tissue coverage in a patient presenting with femur-infiltrating leiomyosarcoma. **(A)** Pre-OP MRI of the left knee **(B)** Resected distal femur with tumor free margins **(C)** Intraoperative situation with implanted modular tumor prosthesis after tumor resection. **(D)** Post-operative esthetic outcome in the 6 months follow-up.

Case 4 (Figure 4) demonstrates a 52-year old male patient presenting with G3 leiomyosarcoma of the distal femur (G2/G3, pT2b, L0, V0, Pn0). Oncological resection included the distal femur and knee joint, which was then reconstructed with a modular endoprosthetic device by the orthopedic surgeons. The resulting soft-tissue defect of 16.5 × 5.5 cm was covered with a free ALT flap from the contralateral thigh performed by the plastic surgeons.

## Discussion

We retrospectively analyzed 803 patients treated for soft-tissue and bone sarcoma by the Department of Plastic and Hand Surgery in the observed study period. Within the last 20 years, one-quarter of all sarcoma patients (24.4%) required free tissue transfer after oncosurgical resection. Microsurgical reconstruction was necessary for 48 patients with soft tissue, bone sarcoma, and soft-tissue sarcoma infiltrating bones to restore soft tissue defects or preserve limb functionality. We demonstrated demographic data for this cohort in line with previously published literature. Patients treated with microsurgical techniques for bone affecting sarcomas were evenly distributed between both sexes with a slightly male predilection (1: 1.3 ratio) as reported elsewhere (9). The age distribution of the presented study population is also in line with the available literature on soft-tissue sarcoma (9, 10). Patients who underwent microsurgical coverage for soft-tissue sarcoma affecting the bone and bone sarcoma were slightly younger compared to the overall study population (54.2 ± 21.7 years vs. 58.6 ± 18 years, mean age ± SD).

Besides the extent of the tumor, its localization, and histologic subtype, R0-resection is of utmost importance for local tumor control and mainly predicts the overall survival (11–13). Histopathological evaluation revealed that only 50% of all cases were made up by three sarcoma entities (osteosarcoma, pleomorphic sarcoma, and fibrosarcoma), while the other 50% were divided into another seven subtypes reflecting the inhomogeneity of soft-tissue and bone sarcoma (14). The evaluation of resection margins for the subgroup showed microscopically tumor-free margins in 45 of 48 cases (93.8%). Five-year survival measurement was applicable for 30 patients, of which one patient died due to progressive high-grade liposarcoma, infection, and sepsis. In 620 patients with soft-tissue and bone sarcoma, primary or local defect coverage was possible, compared to 21 cases in which the affected limb had to be amputated (*p* = 0.058). These patients showed local recurrence in 18.2% of all cases (113 cases). The anatomical pattern of cancer localization in both groups showed a predominance of the lower limbs for all sarcoma subtypes combined. With 66.7% of the cases, the analyzed subgroup of soft-tissue sarcomas affecting the bone and bone sarcomas revealed an overrepresentation of the lower extremity compared to the available literature (1). Notably, the often tricky presentation and the relative rareness of soft-tissue sarcomas impede early diagnosis, particularly in the lower extremities, where soft-tissue swelling stays longer unrecognized (15).

We based flap selection on individual parameters such as tumor and defect size, localization, and peri-/intraoperative patient-related conditions, such as patient positioning. If possible, aesthetic principles were also considered. The overall number of major complications was low, with a flap loss rate of 4% and a revision rate of 10.4%. The anterolateral thigh (ALT) flap was the preferred option for soft-tissue defect reconstruction (41.7%). The relative preference in the analyzed cases toward the ALT free flap was due to its high vascular reliability and consistency (16), and the superb experience with this flap in our team (17). The ALT also resembled the plastic surgical principle to replace “like with like” tissue in most cases, resulting in excellent aesthetics at the cost of minimal donor site defects (17, 18). Also, convenient planning for this reconstructive option makes the ALT flap an excellent choice for limited defect sizes. Due to highly trained and organized microsurgeons, the complex reconstruction of the sarcoma-related defects did not prolong hospital stay, which is in line with previously published data (19).

Our findings, as well as extensive retrospective analysis by other groups, undermine that wide excision supersedes compartmental excision (6, 20). Thus, the presented data supports confidence in the efficacy and safety of limb-sparing surgery. Furthermore, the reported 5-year survival rate for the subgroup of complex microsurgical reconstruction (96.7%) is in line with previously published data. Here, limb preservation has shown no disadvantage for the overall survival compared to amputation of the affected (21–23). However, limb-sparing resections might show inferior local control. With 12.5% of the analyzed subgroup cases developed local recurrence, and 25% presented with distant metastasis, patients treated in our department showed risk for tumor recurrence in line with current literature (24). Still, applying plastic surgical principles facilitates limb preservation with the restoration of function even in large tumors. Thus, the utilization of microsurgical reconstruction in sarcoma defects represents a reliable and safe option (25, 26), and is favorable over local options in regards of complication rates and functional outcome (26, 27). By following oncosurgical principles, our results with low amputation rates resembled data published elsewhere (6).

Gutierrez et al. demonstrated the advantages in overall survival and limb-sparing of soft-tissue sarcoma patients treated in high-volume centers over medical centers with a low number of cases (4). The availability of an experienced team of plastic surgeons to guarantee limb-preservation and safe tumor margins and to reduce amputation rates seems vital. However, amputations in complex sarcoma situations involving the limb remains a therapeutic option, and limb-sparing surgery must not be forced at the cost of unsafe tumor margins as limb salvage is only the secondary goal of tumor surgery. Thus, a multidisciplinary tumor board is mandatory to optimize oncological treatment and discuss surgical treatments (28). In critical cases, the impact of amputation on quality of life has to be considered and weigh against declining advantages over limb-spare surgery (29). Surgical treatment should be interdisciplinary in cases where primary instability is inevitable, or resection extent may lead to secondary fracture. Soft-tissue sarcoma patients present initially most often in low volume centers (5), which increases the proportion of previously operated patients and can reduce the operative options for safe tumor resections in the centers, hence creating the requirement for microsurgical defect coverage solutions.

## Conclusion

Stable oncological outcomes with satisfactory functional results and limb preservation can be achieved even for large sarcoma involving bony tissue if oncological principles for resection are respected and reconstruction is performed according to plastic surgical principles. To handle often large resection-related defects in soft tissue and bone, attending surgeons should provide microsurgical techniques. The heterogeneity and complexity of sarcoma demand an interdisciplinary treatment approach provided by high-volume sarcoma centers.

## Data Availability Statement

The datasets analyzed in this manuscript are not publicly available. Requests to access the datasets should be directed to johannes.zeller@uniklinik-freiburg.de.

## Ethics Statement

This study was approved by the ethic committee of the University of Freiburg Medical Center (Nr.: 434/19) and conducted in accordance with the declaration of Helsinki.

## Author Contributions

JZ conducted main part of the analysis and authored the manuscript with JK. JK authored the manuscript with JZ. DB and OW contributed to the authoring of the manuscript. GS, DD-A, and GH contributed to the interpretation of data, authoring, and final approval of the manuscript. SE planned the analysis, contributed in main parts to the authoring of the manuscript, and interpreted the data.

### Conflict of Interest

The authors declare that the research was conducted in the absence of any commercial or financial relationships that could be construed as a potential conflict of interest.
